# Ipsilateral traumatic floating elbow and floating forearm in adults; A case report

**DOI:** 10.1016/j.ijscr.2025.111627

**Published:** 2025-07-08

**Authors:** Atef Awad, Basel Masoud, Mahmoud Gaber, Hany Elhalafawy, Amro Awad, Elsayed Negm

**Affiliations:** a Madinat Zayed Hospital, Abu Dhabi 10028, United Arab Emirates; b Al-Azhar University, Al Hussain University Hospital, Cairo 66600, Egypt; c Tanta University, 66600, Egypt; d Tanta University Hospital, Tanta 66200, Egypt

**Keywords:** Case report, Floating elbow, Floating forearm, Monteggia variant fracture-dislocation, Double floating

## Abstract

**Introduction:**

This case report describes a rare and complex upper limb injury involving ipsilateral traumatic floating elbow and floating forearm in a 35-year-old male. The patient suffered fractures of the left distal third humerus, olecranon, and both forearm bones, along with anterior radial head dislocation and radial nerve injury due to a pump explosion.

**Materials and methods:**

Open reduction and internal fixation of all fractures in a single operation was done with radial nerve exploration.

**Results:**

Post-operative care and rehabilitation led to successful fracture healing, near-full range of motion, and recovery of radial nerve function by 14 weeks postoperatively.

**Conclusion:**

This case highlights the importance of prioritizing injury management in complex upper limb trauma and demonstrates that favorable outcomes can be achieved even in severe polytraumatized limb injuries thought to be the first case in the literature.

## Highlights

• Double floating injury consisting of ipsilateral floating forearm and floating elbow ⁠with pre-operative radial nerve palsy
• ⁠Surgical management done with plates fixations of all injuries with radial nerve exploration
• ⁠Clinical and radiological amazing outcomes had been achieved 3 months post operatively with radial nerve recovery

## 1 Introduction

The term “floating elbow” refers to the simultaneous occurrence of ipsilateral humerus and forearm fractures, resulting in an unstable intermediate articulation. This condition is relatively uncommon. Stanitski and Micheli first coined the term to describe this injury pattern in children, but it has since been extended to adults with similar fracture patterns. Typically caused by high-energy trauma, this injury is often associated with severe soft tissue damage, open fractures, and potential neurovascular compromise [1].

Floating forearm injury is an injury involving a Monteggia variant fracture with an ipsilateral distal both bone forearm fracture in an adult patient [2]. Monteggia variant fracture is a Monteggia fracture with an associated radial head, coronoid fracture, or complex pattern of injury [3].

Monteggia fractures are inherently unstable, making early recognition and prompt open anatomical reduction with internal fixation essential. However, these fractures remain challenging, often leading to complications, suboptimal functional outcomes, and the potential need for additional surgical intervention [4].

We are presenting a very rare case of devastating upper limb injury consisting of floating elbow with ipsilateral floating forearm. The patient also had preoperative radial nerve injury presented with wrist drop. To our knowledge we did not find a description of such injury (ipsilateral floating elbow and floating forearm) in adult patients reported in the literature before.

## 2 Case presentation

A 35-year-old gentleman works as a manual worker in a pump station. The mode of trauma pump was explosion injury that affected mainly his left upper limb. Clinically, patient was hemodynamically stable, polytraumatized left upper limb with left wrist drop, left upper limb vascularity was fine with marked swelling with healthy skin (no skin plister) and no compartment syndrome.

**Fig. 1:**
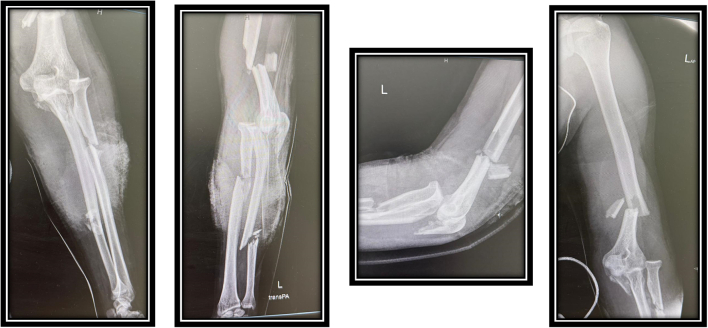
Pre operative X-Rays.

**Fig. 2:**
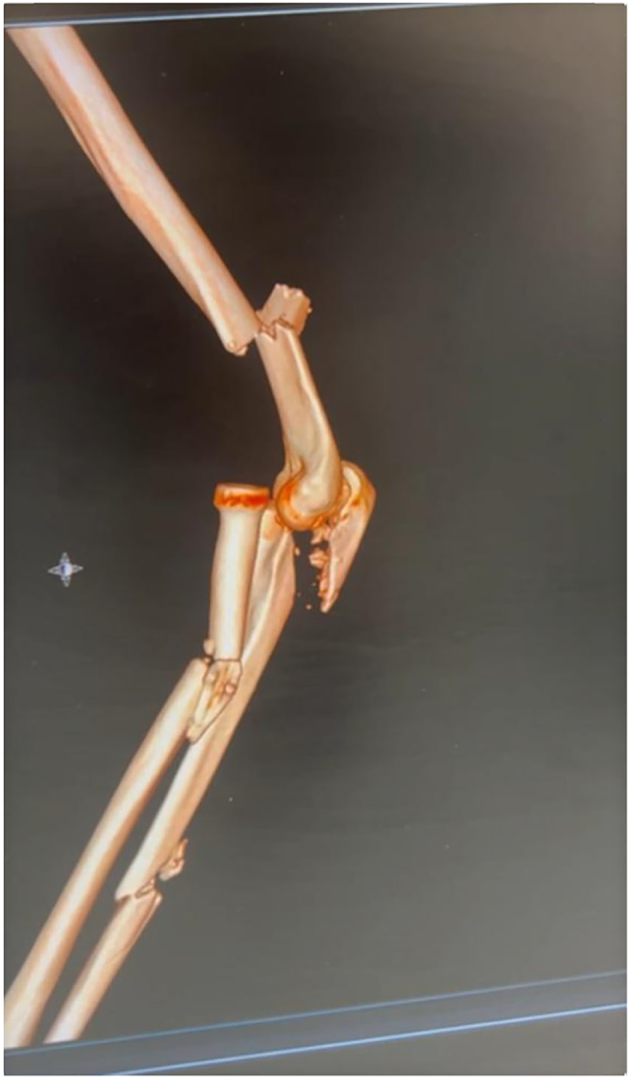
Pre operative 3D scan of the injury.

Radiological studies showed fractures of left lower third humerus, oblique fracture of olecranon with anterior radial head dislocation with radius and ulna diaphyseal fractures as shown in Fig. 1, Fig. 2.

**Fig. 3:**
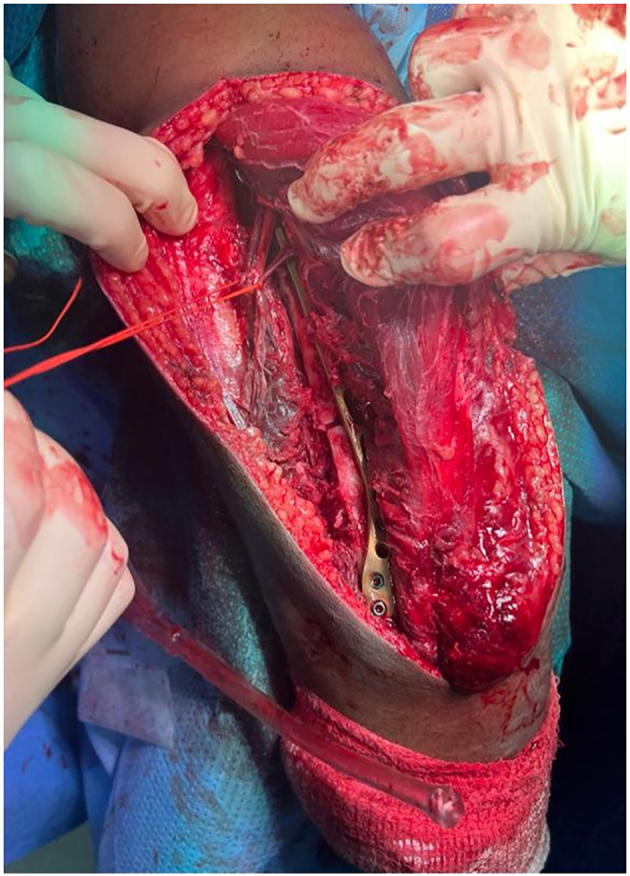
Triceps sparing with radial nerve exploration.

The patient has been prepared for open reduction and internal fixation of all fractures in the same setting. Under general anesthesia, with lateral decubitus position we started with fracture humerus open reduction and internal fixation via posterior triceps sparing approach with radial nerve exploration that had been found to be intact. Humeral fixation was done using anatomical posterolateral distal humerus plate (3.5 LCP DePuy Synthes). After radial nerve exploration it was found intact, suggesting its injury pattern was mostly neuropraxia as shown in Fig. 3. Wound irrigation and closure in layers with good hemostasis,then the patient was turned into supine position, open reduction and internal fixation of olecranon fracture using anatomical olecranon plate (3.5 LCP DePuy Synthes), successful closed reduction of anterior radial head dislocation was done. Then, through the volar Henry approach, open reduction of proximal radial shaft fracture with internal fixation using locked small (3.5 LCP DePuy Synthes). Finally, open reduction and internal fixation of ulnar shaft fracture was done using small, locked (3.5 LCP DePuy Synthes).

**Fig. 4:**
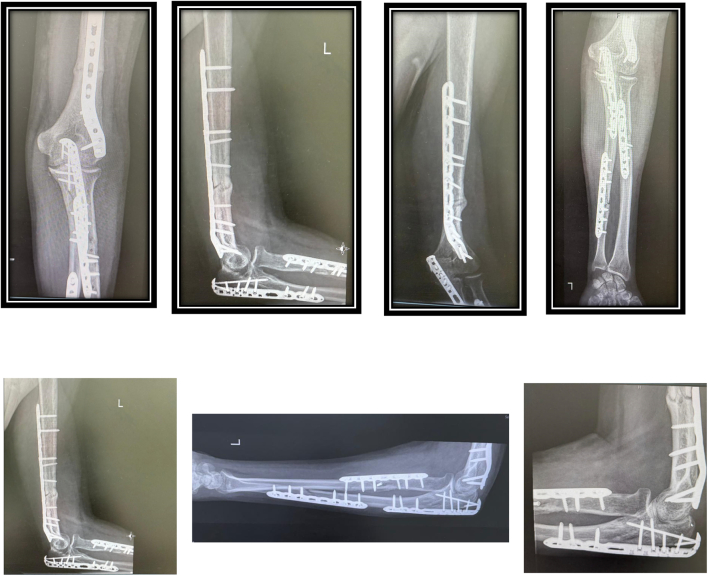
Post operative X-Rays.

At the end of procedures intraoperatively, Range of motion of elbow and forearm rotation had been checked under image intensifier and was found stable with full range of elbow flexion, extension- forearm pronation-supination with intact distal vascularity as shown in Fig. 4.

**Fig. 5:**
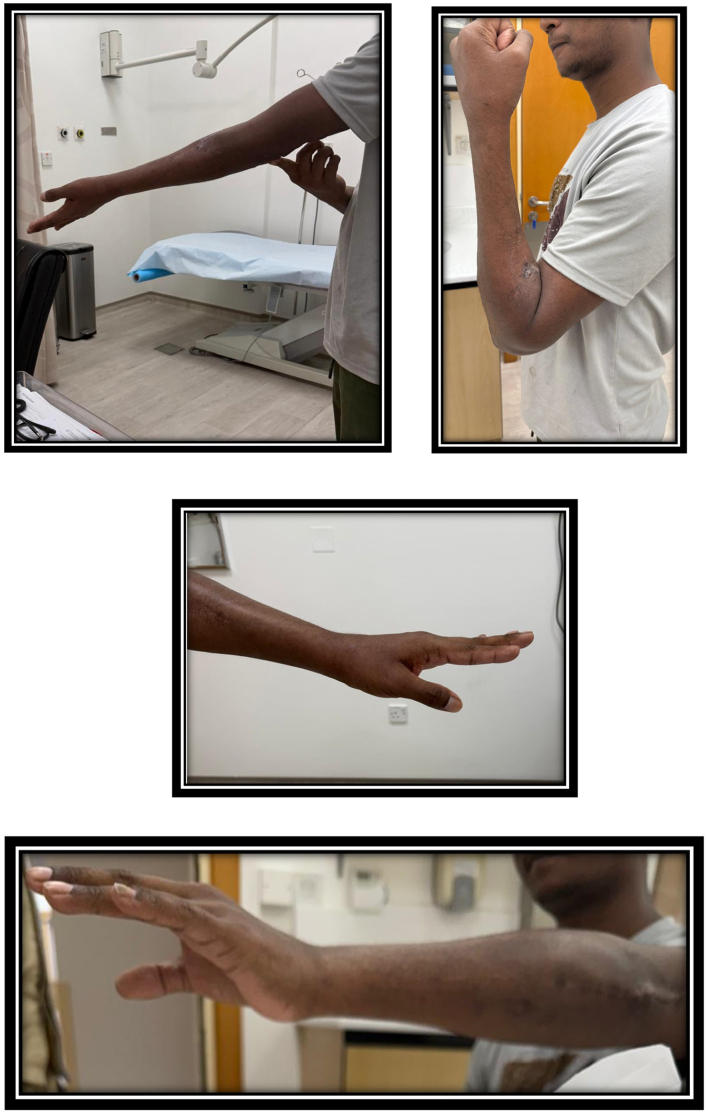
Postoperative clinical photos showing elbow and forearm range of motion with radial nerve recovery.

Postoperative care and rehabilitation protocol: The skin was intact without any signs of infections, dhysince or plisters. Above elbow back slab for 2 weeks with cock-up splinting for wrist drop for 8 weeks. Gradual range of motion exercises of elbow and forearm was started after the 2nd postoperative week. Radial nerve recovery with active wrist dorsiflexion had been observed 9 weeks after surgery. Radiological healing of all fractures was observed at 14 weeks postoperatively, with near full elbow range of motion flexion- extension arc. Forearm supination-pronation arc was 100 degrees. Active wrist dorsiflexion and all fingers extension were as shown in Fig. 5.

## 3 Discussion

Floating elbow injuries in adults are rare, complex conditions involving simultaneous fractures of the humerus and forearm bones. These injuries often result from high-energy trauma, such as falls or traffic accidents, and are frequently associated with complications like nerve damage, vascular injury, and infection [1,5,6].

The description of floating forearm injury in adult had been reported recently in the literature consisting of Monteggia variant fracture associated with ipsilateral distal ulna and radius fractures [2].

In this paper we are reporting a rare case of a new description of ipsilateral double floating (floating elbow and floating forearm) with radial nerve injury.

The rarity of this case is being an ipsilateral combination of floating elbow (fracture humeral shaft with the diaphyseal both bones of forearm fractures), plus monteggia fracture dislocation.

Monteggia fractures are highly unstable and early recognition of the injury with open anatomical reduction with internal fixation is crucial [7].

While we represent this case we recommend the arrangement of priorities of injuries management.

We started first with humerus shaft fixation and radial nerve exploration followed by olecranon fixation and closed reduction of the radial head followed by fixation of the diaphyseal ulnar and radial shaft fractures.

The prognosis of Monteggia fracture dislocation in adults is generally favorable with appropriate treatment, but it can be influenced by several factors. Recent studies indicate that the mean time for fracture union is approximately 4.1 months [8] [9].

Provided that the availability of skilled surgeon, anesthesia and operating room equipment, Our recommendations in such cases is to be done in a single stage. The total time consumed in this case was about 4.5 h.

We think that what we have achieved in this case was magnificent achievement, Contrary to the usual bad expectations in such complex injuries of severe explosion injury with polytraumatized upper limb (Double Floating upper limb injuries, Floating Elbow plus Floating Forearm in adult with radial nerve injury).

## 4 Clinical message

We are representing a rare clinical scenario of a case that we thought to be the first case in the literature of double floating (ipsilateral floating elbow and floating forearm) with its management till full radiological and functional recovery.

## 5 Conclusion

We reported unusual and rare occurrence of ipsilateral Double floating elbow and floating forearm injury in adults. We think this is the first case to be reported in the literature with detailed description of its management including preoperative assessment, intraoperative approach and postoperative protocols with full recovery of radial nerve palsy.

## 6 Methods

The work has been reported in line with the SCARE criteria [10] [11].

## Learning point of the article

Highlights a complicated poly traumatized upper limb with rare injury of double floating forearm and floating elbow thought to be the first case in the literature describing this injury. Management done with satisfactory clinical and radiological results.

## Consent

Written informed consent was obtained from the patient for publication and any accompanying images. A copy of the written consent is available for review by the Editor-in-Chief of this journal on request.

## Ethical approval

Subject: Research Ethics approval of the study Title: “Ipsilateral traumatic floating elbow and floating forearm in adults; a case report.”

The approval is given based on the understanding that the Pl and the research team complies with the applicable guidelines and regulations governing conduct of non-human subjects'.

**The copy of ethical approval form is available upon request.

## Sources of funding

Self-funded (N/A).

## Research registration

Not applicable in our case (our case report not detailing a new surgical technique or new equipment/technology).

## Declaration of competing interest

No conflicts of interest (N/A).
